# Gut microbiota in esophageal cancer: mechanisms, clinical implications, and therapeutic potential

**DOI:** 10.3389/fmicb.2026.1955627

**Published:** 2026-10-05

**Authors:** Yonghao Li, Lu Zhou, Ruixue Huang, Yu Wang, Haibin Zhao, Meijuan Sun, Yushuang Zhang, Zhongbing Wu, Jing Li

**Affiliations:** 1 College of Integrated Chinese and Western Medicine, Hebei Medical University, Shijiazhuang, China; 2 Department of Medical Oncology, Gu'an County People's Hospital, Langfang, Hebei, China; 3 Department of Traditional Chinese Medicine, The Fourth Hospital of Hebei Medical University, Shijiazhuang, China

**Keywords:** biomarkers, causal inference, esophageal cancer, gut microbiota, gut-esophageal axis, microbiota-targeted intervention

## Abstract

Esophageal cancer (EC) is a highly prevalent and aggressive gastrointestinal malignancy. Late diagnosis and heterogeneous responses to chemoradiotherapy and immune checkpoint inhibitor (ICI) therapies severely challenge clinical management. Accumulating evidence suggests that gut microbiota dysbiosis influences esophageal mucosal malignant transformation, tumor progression, and therapeutic outcomes through the gut-esophageal axis. However, most existing research remains correlational. To bridge this gap, this review critically synthesizes the dysbiosis signatures of EC and establishes a five-tier evidence grading framework to rigorously distinguish causal mechanisms from preclinical inferences. This microbial regulation is predominantly driven by metabolite-mediated signaling, remodeling of the tumor immune microenvironment (TME), intestinal barrier disruption, and oncogenic pathway activation. We functionally classify EC-associated dysbiosis into pro-tumorigenic, anti-tumorigenic, and context-dependent taxa, emphasizing a multidimensional ecosystem perspective. Furthermore, we evaluate the utility of gut microbial signatures as predictive biomarkers for neoadjuvant therapy and prognosis. Microbiota-targeted intervention strategies, including fecal microbiota transplantation (FMT), microecological preparations, and Traditional Chinese Medicine (TCM), have entered clinical exploration as adjunctive approaches to optimize treatment efficacy. Finally, we address current translational barriers and propose future directions, emphasizing multi-omics integration, spatial transcriptomics, and artificial intelligence (AI). This provides an objective, mechanistically grounded framework for advancing microbiota-guided personalized oncology in EC.

## 1 Introduction

Esophageal cancer (EC) is an aggressive gastrointestinal (GI) malignancy with a poor global prognosis. According to GLOBOCAN 2022, EC ranks 11th in incidence and 7th in cancer-related mortality, accounting for 510,716 new cases and 445,129 deaths annually (Bray et al., 2024). The two main subtypes are esophageal squamous cell carcinoma (ESCC) and esophageal adenocarcinoma (EAC). ESCC accounts for greater than 85% of global cases, nearly 80% of which occur in Southeastern and Central Asia, with China representing a high-incidence region (Thrift, 2021).

Due to subtle early symptoms, over two-thirds of patients present at locally advanced or metastatic stages, which severely restricts curative options (Yang et al., 2024). Despite recent multidisciplinary advances involving minimally invasive surgery, chemoradiotherapy, and immune checkpoint inhibitors (ICIs) (Yang et al., 2024), outcomes remain limited. This is primarily attributable to inter-subtype (ESCC vs. EAC) and intra-subtype molecular heterogeneity, which frequently leads to unpredictable responses to neoadjuvant therapy, immunotherapy, and targeted agents, thereby complicating clinical decision-making and hindering personalized treatment (Deboever et al., 2024).

Moreover, region-specific environmental influences on the esophageal and gut microbiota remain poorly understood. Heterogeneity in microbial signatures, coupled with interactions between the tumor microbiome and the tumor microenvironment (TME), further contributes to variable therapeutic responses (Jiang et al., 2025). Collectively, these knowledge gaps underscore the need for innovative strategies in EC prevention, early detection, and precision management.

Gut microbiota dysbiosis is recognized as a critical modulator of ESCC initiation and progression via the gut-esophageal axis (Baba et al., 2025). Metagenomic advances have facilitated mechanistic studies of microbiota-host crosstalk (Jin et al., 2022). Accumulating evidence indicates that gut microbiota and their metabolites influence tumorigenesis, progression, and treatment responses by remodeling the immune microenvironment and regulating immune cell differentiation (Elkrief et al., 2025; Javdan et al., 2020; Ning and Hong, 2024).

Specifically, baseline microbial signatures profoundly shape pathological responses to neoadjuvant immunochemoradiotherapy (nICRT) and anti-PD-1 efficacy (Zhou et al., 2026; Liu et al., 2024). Rationally modulating this ecological network has therefore emerged as a promising frontier for therapeutic optimization (Xu et al., 2024; Song et al., 2024; Li J. et al., 2025; Cheung et al., 2020; Chiu et al., 2023). This review critically synthesizes the roles of the gut microbiota in EC pathogenesis and therapy. First, we functionally classify EC-associated dysbiosis into pro-tumorigenic, anti-tumorigenic, and context-dependent taxa. Second, we evaluate gut microbial signatures as predictive biomarkers for neoadjuvant therapy and prognosis. Finally, we assess the translational potential of targeted interventions, such as probiotics, fecal microbiota transplantation (FMT), and Traditional Chinese Medicine (TCM), to overcome resistance. This assessment provides an objective framework for integrating microbial ecology into precision immuno-oncology.

## 2 Theoretical basis of gut microbiota in EC pathogenesis

### 2.1 Biological characteristics and physiological functions of gut microbiota

The human gut microbiota represents the largest and most diverse microbial compartment in the body, dominated by the phyla Firmicutes and Bacteroidetes (Liu et al., 2022; de Vos et al., 2022). This dynamically regulated ecosystem performs essential metabolic and immunomodulatory activities, which are continuously influenced by host genetics, diet, and clinical interventions (de Vos et al., 2022). Fecal sampling remains the standard for non-invasive clinical research due to its reproducibility in large cohorts. However, these non-invasive samples frequently fail to fully recapitulate the mucosa-associated microbiota interacting with the intestinal epithelium (de Vos et al., 2022). Under physiological conditions, bidirectional microbiota-host crosstalk maintains homeostasis via interconnected metabolic, immune, and barrier functions. Commensal microbes metabolize complex carbohydrates and primary bile acids into bioactive mediators, primarily short-chain fatty acids (SCFAs) and secondary bile acids. These metabolites serve dual purposes: they regulate host energy homeostasis and actively reinforce intestinal epithelial tight junctions to prevent bacterial translocation. Concurrently, commensals educate the adaptive immune system, maintain mucosal tolerance, and exert baseline anti-inflammatory effects by inhibiting nuclear factor kappa B (NF-κB) signaling (Liu et al., 2022; de Vos et al., 2022).

Conversely, gut microbiota dysbiosis compromises this physical barrier and disrupts metabolic-immune homeostasis. Aberrant metabolite profiles and pathogenic overgrowth activate pro-inflammatory cascades in the GI mucosa, driving the continuous release of reactive oxygen species, cyclooxygenase-2 (COX-2), and pro-inflammatory cytokines including TNF-α, IL-6, and IL-1β (Liu et al., 2022; de Vos et al., 2022). This sustained inflammatory signaling establishes a pro-tumorigenic microenvironment that actively facilitates genomic instability, aberrant cellular proliferation, and subsequent immune evasion, ultimately accelerating GI carcinogenesis (Van Hul et al., 2024; Fan and Pedersen, 2021).

### 2.2 Research basis for the association between gut microbiota and EC

As an upper GI malignancy, EC warrants investigation regarding its relationship with the distal gut microbiota. While microbiome research in lower GI malignancies, particularly the validation of *Fusobacterium nucleatum*-driven pro-tumorigenic effects in colorectal cancer (CRC), provides a foundational reference (Scott et al., 2019; Li et al., 2023; Baba et al., 2017), current EC research has predominantly focused on the local oral-esophageal indigenous microbiota. The local pro-inflammatory activities of commensal taxa, such as *Porphyromonas gingivalis* and specific *Streptococcus* subsets, have been preliminarily elucidated (Li et al., 2023; Baba et al., 2017). However, the molecular mechanisms by which the distal gut microbiota regulates EC initiation and progression require further characterization.

EC is closely linked to disrupted mucosal homeostasis. Emerging evidence indicates that distal gut microbes exert systemic regulation via the gut-esophageal axis (Baba et al., 2025; Zhou et al., 2021). Rather than direct local colonization, this inter-organ crosstalk operates predominantly through distal secretome-induced carcinogenesis (Weh et al., 2024). In this model, translocated bacterial components and microbial metabolites enter the systemic circulation to target the esophageal mucosa, simultaneously remodeling the systemic immune microenvironment and sustaining chronic low-grade inflammation (Baba et al., 2025; Li et al., 2023; Zhou et al., 2021). To date, the precise molecular circuitry governing this axis, along with the specific taxa driving these distal effects, has not been fully delineated, representing a primary challenge in EC microbiota research (Li et al., 2023; Baba et al., 2017) (Figure 1).

**Figure 1:**
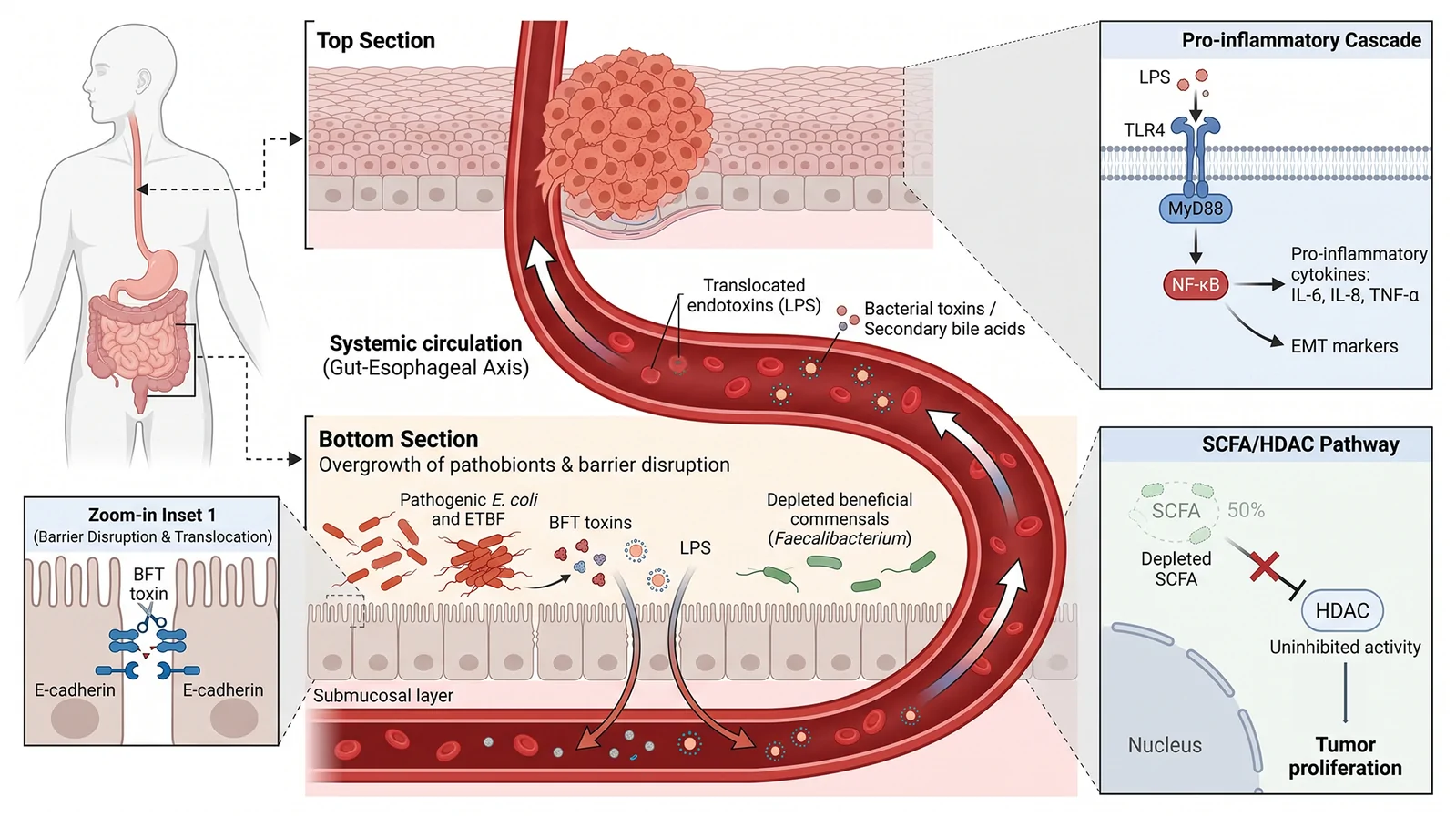
The gut-esophageal axis and distal secretome-induced carcinogenesis in EC. The schematic illustrates how gut dysbiosis **(Bottom)** drives esophageal tumor progression **(Top)**. Key events include BFT-mediated barrier disruption, translocated LPS-induced TLR4/MyD88/NF-κB activation, and SCFA depletion-mediated HDAC overactivation. ETBF, enterotoxigenic *Bacteroides fragilis*; BFT, *Bacteroides fragilis* toxin; LPS, lipopolysaccharide; TLR4, Toll-like receptor 4; MyD88, myeloid differentiation primary response 88; NF-κB, nuclear factor kappa B; EMT, epithelial-mesenchymal transition; SCFA, short-chain fatty acid; HDAC, histone deacetylase; EC, esophageal cancer.

### 2.3 Causal inference and evidence grading in microbiota–EC research

Although numerous studies have reported microbial dysbiosis in EC, most evidence remains correlational. To avoid overstating causality, we systematically apply the Bradford Hill criteria, namely temporality, strength, consistency (reproducibility), specificity, biological gradient (dose-response), plausibility, coherence, experimental evidence, and analogy, to the microbiota-EC literature. Few associations currently satisfy more than a minority of these criteria. In particular, temporality is rarely established because most ESCC studies are cross-sectional, and dose-response data are seldom available. Reverse causation is a major concern: tumor metabolic reprogramming, systemic inflammation, immunosuppression, chemotherapy/radiotherapy, antibiotics, and proton pump inhibitors can all reshape the gut microbiota secondarily, making dysbiosis a consequence rather than a cause of EC or its treatment.

Mechanistic claims must therefore be graded by evidence source. We propose a five-tier framework, ranked from highest to lowest causal strength (A > B > C > D > E): (A) causal evidence from EC-specific *in vivo* models; (B) longitudinal human EC cohort evidence; (C) cross-sectional human EC association; (D) inference from EC *in vitro* systems or non-EC cancer models; and (E) hypothetical mechanisms (Figure 2). This grading is essential because classical Koch's postulates cannot be directly applied to community-level, non-infectious microbiome-cancer interactions (Hosainzadegan et al., 2020), and mechanisms validated in CRC or probiotic/postbiotic models (Feizi et al., 2023, 2024) cannot be assumed to operate in EC. Similarly, oral microbial findings in other cancers (Gholizadeh et al., 2016) should not be extrapolated to the gut-esophageal axis without EC-specific validation.

**Figure 2:**
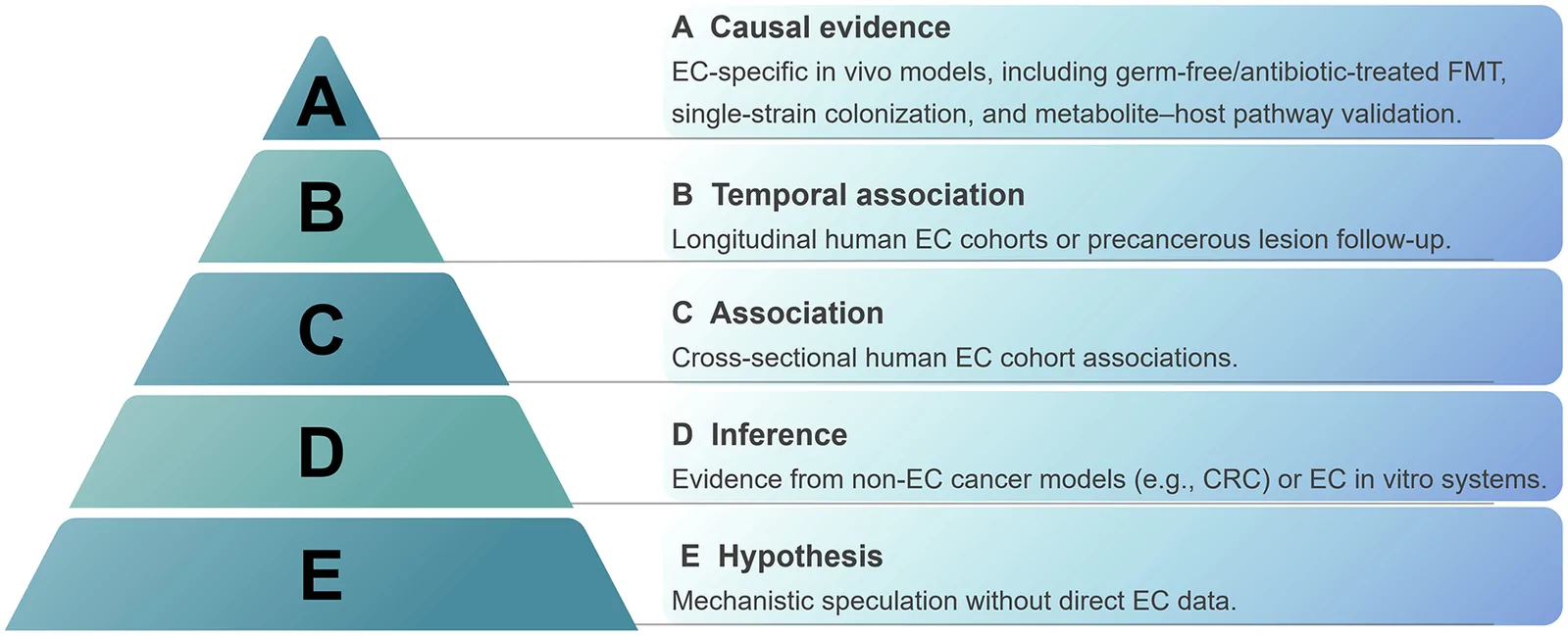
The five-tier evidence grading framework for microbiota-EC research. Evidence is hierarchically stratified from mechanistic hypothesis (Tier E) to direct causal validation (Tier A) to mitigate reverse causation bias and accurately reflect translational readiness.

Recent bidirectional Mendelian randomization studies in EC have provided genetic evidence for a potential causal relationship between gut microbiota and EC risk, while simultaneously revealing reverse causal effects of EC on specific microbial taxa, underscoring the complexity of the causal architecture (Wang K. et al., 2024; Su et al., 2025). Because genetic variants are fixed at conception and largely independent of subsequent disease-related confounders, Mendelian randomization mitigates reverse causation and confounding more effectively than observational association studies.

To disentangle causality, three complementary strategies are required. First, prospective cohorts in EC-endemic regions, encompassing both ESCC- and EAC-endemic areas, should enroll high-risk individuals and patients with precancerous lesions, with long-term follow-up to test whether microbial signatures precede tumorigenesis and independently predict progression. Second, Mendelian randomization should be further applied to test bidirectional causality using genetic variants as instruments for microbial traits. Third, germ-free or antibiotic-treated EC mouse models should be used for FMT experiments, combined with single-strain colonization and metabolite-host pathway analyses, to establish direct causality and identify oncogenic or protective taxa. Until such evidence is available, microbiota-EC associations should be described as correlative or hypothesis-generating, not as established oncogenic mechanisms.

Throughout Sections 3 and 4, each mechanistic statement is followed by an evidence tier in parentheses, formatted as (Tier X; original model: …). Tier A denotes EC-specific *in vivo* causal evidence; Tier B denotes longitudinal human EC cohort evidence; Tier C denotes cross-sectional human EC association; Tier D denotes inference from EC *in vitro* systems or non-EC cancer models; Tier E denotes hypothesis only. This grading applies to mechanistic causality. Clinical trial evidence is separately indicated by study phase [e.g., Phase II randomized controlled trial (RCT)]. A visual summary incorporating this evidence hierarchy is provided in the mechanistic models (Figures 1, 3), and a mechanism-by-mechanism grading is presented throughout the subsequent subsections.

**Figure 3:**
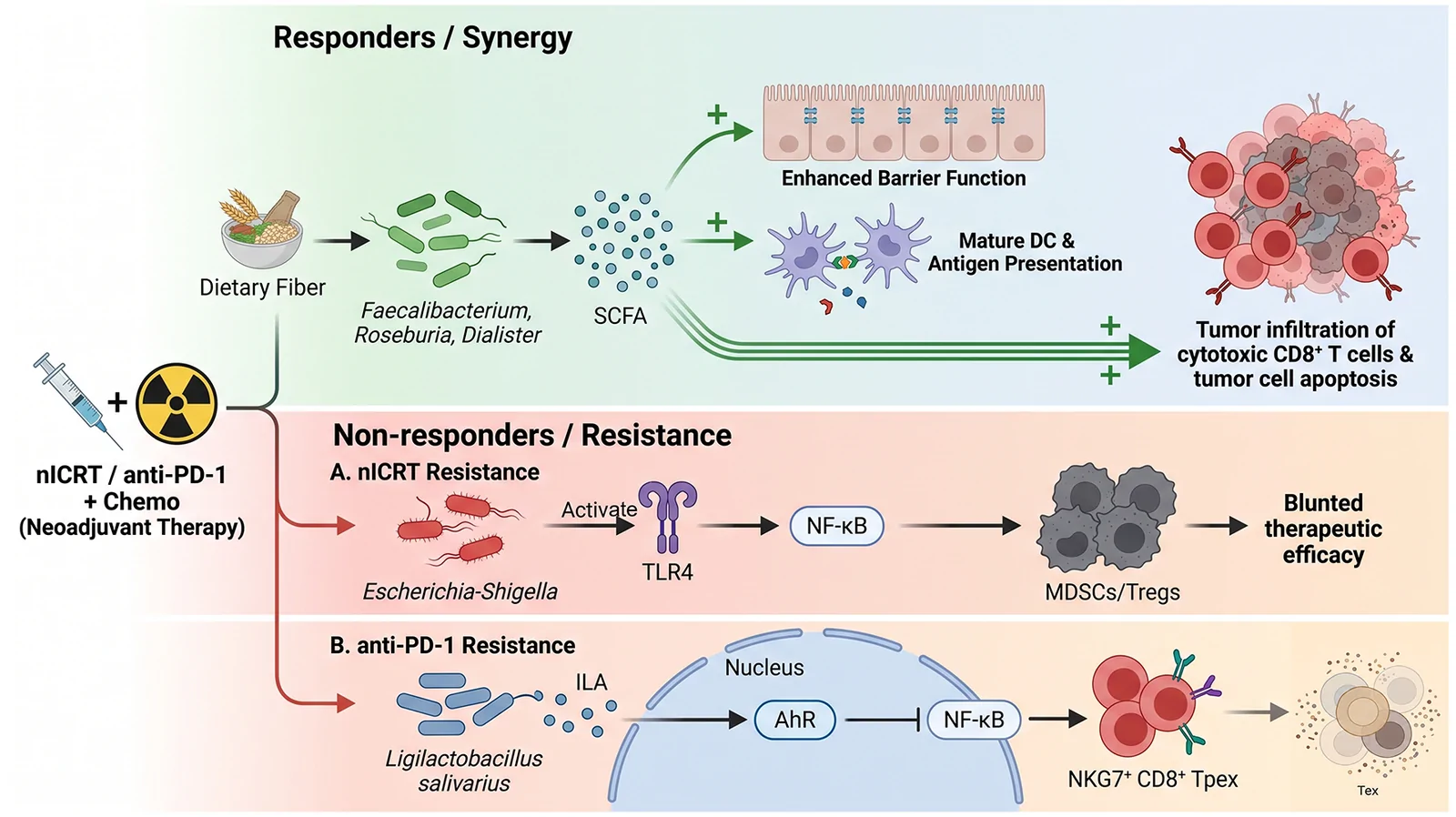
Gut microbiota-mediated mechanisms underlying response and resistance to neoadjuvant therapy in EC. **(Top)** In responders, SCFA-producing commensals enhance barrier integrity and CD8^+^ T cell infiltration. **(Bottom)** In non-responders, resistance is driven by two axes: **(A)** Pathobiont-induced TLR4/NF-κB activation recruits immunosuppressive MDSCs and Tregs; **(B)** *L. salivarius*-derived ILA activates AhR, inducing Tpex exhaustion. DC, dendritic cell; nICRT, neoadjuvant immunochemoradiotherapy; MDSC, myeloid-derived suppressor cell; Treg, regulatory T cell; ILA, indole-3-lactic acid; AhR, aryl hydrocarbon receptor; Tpex, progenitor-exhausted T cell; Tex, terminally exhausted T cell; PD-1, programmed cell death protein 1; SCFA, short-chain fatty acid.

For TLR4/NF-κB, β-catenin, and HDAC-related mechanisms, most current evidence derives from CRC or *in vitro* systems and is therefore graded as Tier D unless EC-specific *in vivo* validation is available.

## 3 Characteristics and core mechanisms of gut microbiota dysbiosis in EC

Most cross-sectional studies comparing the gut microbiota between EC patients and healthy controls have reported compositional differences, with altered abundances of specific genera demonstrating potential diagnostic value. However, results vary across populations, geographical regions, dietary habits, and analytical approaches (Liu et al., 2024; Deng et al., 2021; Cheung et al., 2022a; Shen et al., 2021; Hasuda et al., 2023; Gao et al., 2024; Huang et al., 2024; Ishaq et al., 2021; Wu et al., 2024; Li Y. et al., 2025; Li et al., 2022). Furthermore, most studies utilize binary case-control designs without stratifying patients by disease stage or lymph node metastasis and are constrained by small sample sizes, as most cohorts include fewer than 50 participants (Zhang et al., 2024; Deng et al., 2024). These limitations are characteristic of exploratory studies that prioritize capturing early dysbiosis signals over strict inclusion criteria.

A critical barrier to establishing consensus in EC microbiota research is the severe lack of cross-study comparability. This inconsistency is primarily driven by unstandardized methodologies and uncontrolled clinical confounders, which generate substantial batch effects. First, differences in sample collection methods, such as mucosal biopsies vs. non-invasive fecal sampling, capture distinct ecological niches that cannot be directly compared. Second, variations in DNA extraction kits and sequencing platforms (e.g., Illumina vs. Ion Torrent vs. Nanopore) introduce systemic biases (Fouhy et al., 2016). Third, discrepancies in bioinformatic pipelines, including the use of varying reference databases (e.g., Greengenes, SILVA), clustering algorithms (e.g., QIIME2, DADA2), and inconsistent filtering thresholds for low-abundance taxa, further distort the taxonomic profiles. Fourth, concurrent medication use acts as a major disruptor of data reproducibility. Agents such as antibiotics and proton pump inhibitors exert profound and long-lasting alterations on gut microecology (Weersma et al., 2020), yet these factors are frequently uncontrolled in retrospective case-control cohorts. To achieve molecular reproducibility and eliminate batch effects, future multicenter studies must implement harmonized protocols. Specific recommendations include: (i) unifying the amplification of the 16S rRNA hypervariable regions (e.g., V3–V4) and adopting standardized DNA extraction kits; (ii) using a single sequencing platform where possible and reporting platform-specific biases; (iii) adopting standardized bioinformatic processing pipelines such as QIIME2 with a defined reference database and consistent low-abundance filtering thresholds; (iv) establishing uniform algorithms for batch effect removal; and (v) mandating a defined washout period (e.g., ≥4 weeks) for antibiotics and proton pump inhibitors prior to sampling, with detailed medication records and dietary metadata collected prospectively.

Based on published clinical data evaluating fecal microbial alterations in EC, which predominantly consist of retrospective cross-sectional case-control studies, the observed microbial shifts can be categorized into three recurrent patterns: (1) genera consistently enriched in fecal samples of EC patients compared with age- and sex-matched healthy controls (e.g., *Streptococcus, Bacteroides*, and *Escherichia-Shigella*); (2) genera markedly depleted in EC patients (e.g., *Faecalibacterium* and *Lachnospira*); and (3) genera with conflicting abundance trends across independent cohorts (e.g., *Prevotella*). While preclinical models have demonstrated the pro-tumorigenic roles of enriched taxa and the anti-tumor properties of depleted commensals, these causal relationships remain unconfirmed in human populations. The following sections integrate clinical correlative data and preclinical mechanistic evidence to interpret these microbial patterns. Functional differences frequently exist at the strain or specific effector molecule level rather than the genus level; therefore, the following discussion highlights these refined distinctions where data are available (Table 1).

**Table 1:** Dysbiosis signatures of gut microbiota in esophageal cancer (EC patients vs. healthy controls).

Taxonomic group	Abundance trend in EC	Key strain (s) and core biological effect (concise statement)
*Streptococcus*	↑	*S. anginosus*: Evades innate immunity by degrading NETs, induces mucosal damage via H_2_S and streptolysin S, and triggers pro-inflammatory cytokine storms.
*Bacteroides*	↑ (heterogeneous)	Enterotoxigenic *B. fragilis* (ETBF): Secretes BFT to cleave E-cadherin and activate β-catenin; LPS from diverse strains triggers TLR4/NF-κB crosstalk.
*Escherichia-Shigella*	↑	*pks*^+^ pathogenic *E. coli*: Delivers colibactin inducing DNA double-strand breaks; Shiga toxin impairs secretory IgA and disrupts epithelial integrity.
*Prevotella* spp.	Context-dependent (↑/↓)	Dual role: Degrades mucins via NF-κB/STAT3 (pro-tumor) or produces SCFAs to maintain homeostasis (anti-tumor), dynamically shaped by dietary fiber.
*Faecalibacterium prausnitzii*	↓	Produces butyrate and salicylic acid to inhibit HDAC/NF-κB, upregulate IL-10, strengthen tight junctions, and induce Treg differentiation.
*Lachnospira* spp.	↓	Generates SCFAs to activate AMPK, promote dendritic cell maturation and CD8^+^ T infiltration, while reducing MDSC accumulation in the TME.

Abundance symbols indicate the qualitative shift in relative abundance in fecal samples of EC patients compared with healthy controls based on cross-cohort consensus: ↑, enriched (increased abundance); ↓, depleted (decreased abundance). Quantitative gradations are excluded due to inter-study methodological heterogeneity.

Pro-tumor pathobionts (Streptococcus, ETBF) and protective butyrate producers (Faecalibacterium, Lachnospira) are consistently identified across cohorts; however, the context-dependent role of Prevotella and the lack of species-level resolution remain critical knowledge gaps that limit mechanistic interpretation.

### 3.1 Enrichment characteristics and associated mechanistic evidence of EC-enriched gut microbiota

Consistent enrichment of specific bacterial genera is a primary feature of gut dysbiosis in EC. These taxa act synergistically to disrupt intestinal homeostasis, establish a pro-inflammatory microenvironment, and drive esophageal malignant transformation (Table 2). Three genera have been widely investigated, with accumulating preclinical evidence supporting their roles in EC progression.

**Table 2:** Core mechanisms linking gut microbiota to esophageal cancer pathogenesis.

Mechanism category	Key microbiota/metabolites	Core conclusion (direct mechanistic statement)
Chronic inflammation and immune modulation	LPS, *S. anginosus*, ETBF	Activate TLR4/MyD88 and NF-κB/MAPK/STAT3 cascades, driving excessive IL-1β and TNF-α release, recruiting MDSCs, and suppressing CD8^+^ T cell activity.
Metabolic reprogramming	SCFAs (butyrate, acetate, propionate), lactate	SCFAs inhibit HDAC to exert anti-inflammatory epigenetic effects; exogenous lactate promotes angiogenesis, immunosuppressive polarization, and immune evasion.
Intestinal barrier disruption	BFT (from ETBF), LPS, Shiga toxin	BFT cleaves E-cadherin to increase paracellular permeability; Shiga toxin disrupts epithelial integrity, allowing translocated LPS to trigger systemic low-grade endotoxemia and drive distal secretome-induced carcinogenesis.
Epigenetic and genetic damage	Colibactin, SCFAs, ROS	Colibactin induces DNA double-strand breaks and chromosomal aberrations; SCFAs modulate histone acetylation; ROS promotes oxidative DNA mutagenesis.

Key mechanistic pathways (e.g., SCFA/HDAC, LPS/NF-κB) outlined here are briefly referenced in other tables. Abbreviations: SCFA, short-chain fatty acid; LPS, lipopolysaccharide; HDAC, histone deacetylase; ROS, reactive oxygen species; BFT, *Bacteroides fragilis* toxin.

The pathogenesis converges on NF-κB-driven chronic inflammation and HDAC-mediated epigenetic shifts as central hubs driving distal secretome-induced carcinogenesis; however, the causal hierarchy among barrier loss, metabolic change, and immune suppression remains unproven in dynamic in vivo models.

#### 3.1.1 Streptococcus

Multiple clinical studies have confirmed the enrichment of *Streptococcus* in fecal samples from EC patients, particularly those with ESCC (Deng et al., 2021; Cheung et al., 2022a; Shen et al., 2021; Hasuda et al., 2023; Gao et al., 2024; Huang et al., 2024). Huang et al. (2024) observed a positive correlation between the abundance of *Streptococcus* and *Lactobacillus* in the fecal microbiota of ESCC patients. Both taxa exhibit lactic acid-producing properties. Consequently, the study proposed a hypothesis that gut-derived lactic acid may act on esophageal tumors via the gut-tumor axis, potentially contributing to the formation of a tumor-permissive niche by promoting angiogenesis, metabolic reprogramming, and immune evasion.

The biological effects of lactic acid in the TME are predominantly attributed to endogenous lactic acid produced by tumor cells via the Warburg effect. These effects include promoting neovascularization, inducing immunosuppressive macrophage polarization, and impairing cytotoxic CD8^+^ T cell cytotoxicity (Chen et al., 2025). Whether exogenous lactic acid produced by gut commensal bacteria can cross the intestinal barrier, reach esophageal tumor tissue through the systemic circulation, and achieve functionally effective concentrations lacks direct *in vivo* causal evidence. Therefore, relevant inferences require further validation.

*Streptococcus anginosus* (*S. anginosus*) is a pathogenic strain within the genus associated with ESCC progression. This facultative anaerobe exhibits intestinal mucosal colonization capacity. Preclinical findings reveal that it induces mucosal damage through a sequential cascade comprising epithelial adhesion and colonization, immune evasion, sustained inflammation, and subsequent intestinal barrier disruption and systemic spread (Coker et al., 2018; Kuryłek et al., 2022). Immune evasion is essential for its persistent colonization. It primarily secretes deoxyribonuclease to degrade neutrophil extracellular traps (NETs). This mechanism, observed in pyogenic and periodontal infections, is proposed to facilitate its survival in the intestinal mucosa, while specific strains additionally resist phagocytosis via capsular polysaccharides. Furthermore, certain β-hemolytic streptococcal strains (groups A, B, C, and G) harbor genes encoding superantigens. These secreted superantigens can activate a large proportion of T cell clones in an antigen-independent manner, triggering a pro-inflammatory cytokine storm and systemic immune dysregulation (Spaulding et al., 2013).

Additionally, *S. anginosus* causes dual mucosal injury. It activates intracellular signaling pathways to induce the release of IL-1β and TNF-α. Concurrently, it produces hydrogen sulfide (H_2_S) through L-cysteine desulfhydrase-mediated amino acid metabolism and secretes streptolysin S to directly damage epithelial cells, jointly aggravating tissue damage (Coker et al., 2018; Kuryłek et al., 2022). In *in vitro* models of respiratory co-infections, interbacterial cooperation with *Pseudomonas aeruginosa* and α-enolase-mediated plasminogen activation further enhance its virulence and invasiveness. This interaction provides a reference for understanding its pathogenic potential in the intestinal tract (Kuryłek et al., 2022).

#### 3.1.2 Bacteroides

Enrichment of *Bacteroides* has been reported in gut microbiota studies of both ESCC and EAC; however, inter-cohort heterogeneity limits its classification as a conserved dysbiosis signature, and its proposed role in EC progression requires further validation (Cheung et al., 2022a; Hasuda et al., 2023; Huang et al., 2024; Ishaq et al., 2021; Wu et al., 2024; Li et al., 2022; Hao et al., 2022). The underlying mechanism is hypothesized to involve intestinal barrier disruption, inflammatory microenvironment remodeling, and subsequent epithelial malignant transformation, in which enterotoxigenic *Bacteroides fragilis* (ETBF) and its secreted *B. fragilis* toxin (BFT) act as primary effector molecules.

In CRC models, BFT, the primary virulence effector of ETBF, specifically cleaves the extracellular domain of E-cadherin, a structural component of intestinal epithelial adherens junctions. This cleavage increases epithelial paracellular permeability, facilitates bacterial translocation across the mucosal barrier, and initiates downstream inflammatory cascades (Deng et al., 2024). Beyond barrier disruption, BFT drives malignant transformation via oncogenic signaling in CRC models: E-cadherin shedding releases and activates β-catenin, which translocates to the nucleus to upregulate oncogene expression (Tier D; original model: CRC). Whether this BFT–E-cadherin–β-catenin axis operates in EC remains untested. Concurrently, sustained BFT-induced interleukin-8 (IL-8) secretion perpetuates a chronic inflammatory milieu that supports tumor progression (Patrick, 2022; Abate et al., 2022). However, these mechanistic insights regarding ETBF and its secreted BFT are predominantly derived from CRC models.

ETBF also reshapes intestinal immune homeostasis through distinct pathways. Aside from secreted ubiquitin-like proteins that interfere with host signaling via molecular mimicry (Patrick, 2022), its surface capsular polysaccharide A (PSA) mediates bidirectional, context-dependent immunomodulation through a T cell-dependent axis. As demonstrated by Alvarez et al. (2020), PSA is processed and presented to CD4^+^ T cells via major histocompatibility complex (MHC) class II molecules, driving an interferon-responsive gene (IRG) signature. In parallel, PSA stimulation induces the upregulation of co-inhibitory receptors on CD4^+^ T cells, an effect verified to be IFN-γ-dependent. This simultaneous induction of pro-inflammatory cytokines and inhibitory surface receptors confers a mixed phenotype on PSA-responding T cells, indicating that the net immunological outcome is heavily dependent on the local tissue microenvironment (Alvarez et al., 2020).

Furthermore, ETBF-induced epithelial barrier damage permits the translocation of LPS, a cell wall component from diverse Gram-negative gut bacteria, into the systemic circulation. In ESCC cell lines, translocated LPS drives disease progression via the aforementioned TLR4/MyD88/NF-κB axis (Tier D; original model: ESCC cell lines/*in vitro*) (Wu et al., 2024). While these findings support a pro-tumorigenic hypothesis for specific pathogenic *Bacteroides* strains, the genus as a whole comprises numerous commensal lineages with no demonstrated pathogenic potential. This functional heterogeneity underscores the need for strain-level resolution and EC-specific *in vivo* tracking (e.g., utilizing orthotopic models to trace bacterial derivatives like outer membrane vesicles) in future mechanistic investigations.

#### 3.1.3 Escherichia-Shigella

Findings regarding *Escherichia-Shigella* enrichment in ESCC vary across cohorts, although several studies report elevated fecal abundance in patients (Zhou et al., 2026; Gao et al., 2024; Huang et al., 2024). The *Escherichia-Shigella* taxon resolved by 16S rRNA sequencing is a composite unit lacking species- and strain-level resolution. It encompasses both commensal *Escherichia coli* (*E. coli*) and pathogenic *Escherichia*/*Shigella* subtypes. The pro-tumor virulence traits described below are restricted to pathogenic strains rather than being universal to the entire amplicon-defined group.

Preclinical studies demonstrate that pathogenic *Escherichia-Shigella* drives intestinal mucosal tumorigenesis via sequential barrier disruption, inflammatory activation, and malignant transformation. These pathogens adhere to epithelial cells via fimbriae and deliver effector proteins through the type III secretion system. Two primary virulence factors mediate mucosal defense impairment: LPS, released primarily via bacterial lysis or outer membrane vesicle shedding, and Shiga toxin (Stx), a cytotoxic protein from specific pathogenic subtypes that targets host 28S rRNA to inhibit protein synthesis, inducing necrosis and apoptosis in globotriaosylceramide (Gb3)-expressing epithelial cells. Collectively, these factors compromise epithelial integrity, reduce mucosal mucin production, and suppress secretory immunoglobulin A (sIgA), impairing both physical and immune barriers (Pakbin et al., 2021; Hanson and Weiss, 2024; Havira et al., 2020; Kang et al., 2018).

Released LPS triggers pro-inflammatory responses through the NF-κB pathway and activates mitogen-activated protein kinase (MAPK) signaling to amplify TNF-α and IL-8 secretion *in vitro* (Tier D; original model: *in vitro*), sustaining a pro-tumorigenic inflammatory microenvironment (Pakbin et al., 2021; Kang et al., 2018). Beyond epithelial inflammation, pathogenic *Escherichia-Shigella* remodels intestinal immune homeostasis to facilitate colonization and tumorigenesis. It evades innate immune clearance by modifying outer membrane lipid A to reduce pattern recognition receptor activation and subverts antimicrobial peptide activity via surface charge alterations rather than direct peptide degradation. Furthermore, type III secretion system effectors internalized by mucosal dendritic cells (DCs) perturb the T helper 1 (Th1)/Th17/Treg balance by skewing naive CD4^+^ T cell differentiation toward pro-inflammatory Th1/Th17 lineages and weakening Treg suppressive function. The resulting pro-inflammatory T cell dominance drives the sustained secretion of IFN-γ, IL-6, and interleukin-17A (IL-17A), exacerbating mucosal damage and creating a tumor-permissive milieu. In addition to systemic immune dysregulation, intratumoral colibactin-producing *E. coli* establishes local immunosuppression via lipid metabolic reprogramming. This process reduces CD8^+^ T cell infiltration and IFN-γ production in colonized niches, correlating with tumor recurrence and chemoresistance (de Oliveira Alves et al., 2024).

Beyond barrier injury and chronic inflammation, specific pathogenic *E. coli* strains encode virulence systems that directly drive malignant transformation. Strains harboring the *pks* genomic island produce colibactin, a genotoxin that induces DNA double-strand breaks, chromosomal aberrations, and cell cycle dysregulation to promote genomic instability (Iftekhar et al., 2021). Short-term exposure to *pks*^+^ *E. coli* transforms primary colon epithelial cells into Wnt-independent, persistently proliferating clones via chromosomal instability and disruption of the p53/miR-34 axis, independent of classical adenomatous polyposis coli (APC) mutations (Carlson et al., 2025). The colibactin-associated DNA damage motif AAWWTT (IUPAC code W = adenine or thymine) is enriched in human CRC genomes and overlaps with mutation hotspots of driver genes including APC. This provides population-level etiological evidence (Dziubańska-Kusibab et al., 2020). Additionally, in CRC and other non-EC models, effector proteins from specific pathogenic *E. coli* strains have been shown to activate β-catenin/c-Myc signaling, drive excessive proliferation, inhibit apoptosis, and disrupt DNA repair, further amplifying genomic instability (Tier D; original model: CRC/non-EC models) (Pakbin et al., 2021). Whether these effects occur in EC remains to be determined.

Furthermore, pathogenic *Escherichia-Shigella* outcompetes beneficial commensals through nutrient competition and bacteriostatic metabolites in the intestinal niche, establishing a cycle of dominant colonization, progressive barrier damage, and persistent inflammation. In the context of fecal microbiota alterations in ESCC, the intestinal mucosal injury and systemic inflammation induced by these pathogens contribute to tumor development via the gut-esophageal axis. Translocated LPS from these pathobionts reaches the esophageal mucosa, exacerbating malignant progression directly through systemic inflammatory cascades (Wu et al., 2024). Collectively, these findings support a pro-tumorigenic role for specific pathogenic *Escherichia-Shigella* subtypes, whereas the genus as a whole includes numerous commensal lineages with no confirmed pathogenic or carcinogenic potential.

### 3.2 Controversial roles and influencing factors of dual-regulatory microbiota in EC

#### 3.2.1 The ecosystem framework of Prevotella and Bacteroides

Findings on the abundance of specific taxa, notably *Prevotella* and *Bacteroides*, in ESCC are inconsistent across cohorts (Hasuda et al., 2023; Gao et al., 2024; Huang et al., 2024; Ishaq et al., 2021). Attributing intrinsic and fixed pro-tumorigenic or anti-tumorigenic properties to these genera is fundamentally flawed (Cani, 2018; Simpson et al., 2022). This inconsistency arises because both genera encompass functionally heterogeneous species and strains; for example, ETBF harbors the *bft* gene and produces BFT, whereas commensal *B. fragilis* strains lack this toxin, and *Prevotella copri* can either ferment dietary fiber or degrade mucin depending on substrate availability. Their biological functions must be evaluated within a sophisticated ecosystem framework. The functional output of these bacteria is not static but dynamically modulated by three core dimensions: dietary input, host genetics, and the pathological stage of the disease. These dimensions interact rather than act independently, and their combined effect determines whether these taxa behave as commensals, pathobionts, or opportunistic pathogens.

Regarding dietary input, the availability of specific metabolic substrates governs bacterial functional modes. Saccharolytic *Prevotella* and commensal *Bacteroides* strains ferment complex plant polysaccharides to yield SCFAs, primarily propionate and acetate. These metabolites contribute to host energy harvest and the preservation of intestinal immune homeostasis (Tett et al., 2021). Conversely, under the selective pressure of high-fat or low-fiber diets, these taxa adapt by degrading host-derived mucins (Han et al., 2025; Marzhoseyni et al., 2024). This metabolic shift compromises the mucosal mucus layer, amplifies pro-inflammatory commensal networks, and frequently co-occurs with altered secondary bile acid pools. Notably, diets rich in red meat and saturated fat also promote the expansion of bile-tolerant *Bacteroides* and *Prevotella* species. This expansion increases the production of secondary bile acids (e.g., deoxycholic and lithocholic acid), which directly exacerbate the pro-tumorigenic inflammatory microenvironment without necessitating further delineation of downstream signaling cascades. This alteration reflects systemic ecological disruption rather than direct bacterial bile salt hydrolase activity (Han et al., 2025; Jones et al., 2008; Ridlon et al., 2006). Therefore, dietary patterns should be recorded and stratified in future EC microbiome studies, as they may explain a substantial portion of inter-cohort heterogeneity.

Host genetics provide the baseline immunological context that dictates host-microbe interactions. Genetic polymorphisms in host pattern recognition receptors, including TLR4, NLRP3, and NOD2, as well as immunoregulatory loci such as *IL10* and *HLA* variants, determine the threshold of mucosal immune tolerance. In a genetically susceptible host with impaired immunoregulation, the structural components of *Prevotella* and *Bacteroides* trigger exaggerated mucosal immune responses (Choi et al., 2014). This genetic vulnerability transforms these typical commensals into opportunistic pathobionts that directly establish a chronic inflammatory milieu (Choi et al., 2014). Mendelian randomization studies in EC have begun to explore whether host genetic variants that shape microbial composition also influence EC risk, but whether these interactions are causal remains unresolved (Wang K. et al., 2024; Su et al., 2025). Consequently, host genotype should be considered a key effect modifier rather than a confounding variable in microbiota–EC association studies.

The pathological stage of EC further dictates the exact role of these dual-regulatory microbes. During the early stages of disease, an intact intestinal barrier compartmentalizes these bacteria, allowing them to exert homeostatic effects. In advanced disease stages, tumor-driven systemic inflammation or subsequent treatment-induced mucosal injury severely compromises barrier integrity. This structural disruption permits the translocation of *Prevotella* and *Bacteroides* or their active byproducts into the systemic circulation, exacerbating local Th17-skewed inflammation and accelerating disease progression (Larsen, 2017). In addition, chemotherapy and radiotherapy can further alter the gut microenvironment, selecting for taxa with enhanced stress tolerance and shifting the balance from SCFA-producing commensals to mucin-degrading pathobionts. This stage-dependent shift implies that the same taxon may have opposing associations with EC risk in early vs. advanced disease, which may partly explain the conflicting findings across cross-sectional cohorts.

Therefore, the net impact of *Prevotella* and *Bacteroides* on esophageal carcinogenesis reflects an integrated ecological effect rather than a binary intrinsic oncogenic property (Simpson et al., 2022; Zhou and Zhao, 2026). The classification of these taxa must transition from static abundance metrics to dynamic functional profiling across varying microecological conditions. We propose that future studies adopt a multi-dimensional ecosystem framework that integrates (i) strain-level genomic and functional profiling, (ii) dietary and medication metadata, (iii) host genetic polymorphisms, and (iv) disease stage and treatment history. Such an approach will be essential to determine whether *Prevotella* and *Bacteroides* can serve as reliable biomarkers or therapeutic targets in EC, and to avoid oversimplified interpretations of their dual roles. Given that most current mechanistic evidence for these taxa derives from CRC or *in vitro* models, the roles of *Prevotella* and *Bacteroides* in EC should currently be regarded as Tier C–D evidence (i.e., cross-sectional human association or inference from non-EC models), pending EC-specific *in vivo* validation.

### 3.3 Depletion characteristics and protective mechanisms of anti-tumorigenic microbiota in EC

Corresponding to the enrichment of pathogenic genera, multiple commensal taxa are depleted in EC patients. These taxa maintain gut homeostasis, and preclinical evidence indicates that their anti-tumor activities are mediated by metabolite-driven immune regulation and barrier protection.

#### 3.3.1 Faecalibacterium

*Faecalibacterium*, predominantly represented by *Faecalibacterium prausnitzii* (*F. prausnitzii*), is an abundant core commensal genus in the healthy human gut, accounting for 5% to 6.5% of total fecal bacteria (Martín et al., 2023). This genus is reduced in abundance in fecal samples from patients with ESCC, primarily in East Asian cohorts (Hasuda et al., 2023; Huang et al., 2024; Li Y. et al., 2025; Li et al., 2022). Its roles are mediated by metabolite-driven immunomodulation and the reinforcement of intestinal homeostasis. In preclinical models, mainly CRC and colitis models, butyrate and microbe-derived salicylic acid have been shown to exert anti-inflammatory effects by inhibiting histone deacetylase (HDAC) and NF-κB activity, upregulating the anti-inflammatory cytokine IL-10, and promoting regulatory T cell differentiation (Tier D; original model: CRC/colitis models) (Martín et al., 2023). Furthermore, *F. prausnitzii* secretes anti-inflammatory molecules and extracellular vesicles to upregulate tight junction proteins and protect the mucus layer *in vitro*, strengthening intestinal barrier integrity. By enhancing barrier function and restricting opportunistic pathogen expansion via metabolic dominance, *F. prausnitzii* reduces tumorigenesis risk through mitigation of pathogen-induced chronic inflammation and DNA damage (Lin Y. et al., 2025; Singh et al., 2022; Shi et al., 2025a,b).

Preclinical data reveal its anti-tumor potential across various cancer models through mechanisms including the suppression of Janus kinase (JAK)/STAT signaling and the induction of cancer cell apoptosis (Shi et al., 2025b). Butyrate modulates tumor metabolism via G-protein-coupled receptor 109a (GPR109a) and enhances natural killer (NK) cell-mediated immune surveillance in preclinical settings (Singh et al., 2022). Most of these anti-tumor mechanisms have been characterized in CRC and colitis models, whereas direct experimental validation in ESCC remains limited. The role of transforming growth factor-beta (TGF-β) signaling in this context is dual. Butyrate activates TGF-β to support epithelial repair under physiological conditions. Conversely, TGF-β promotes EMT during advanced tumor progression (Shi et al., 2025b). This duality suggests that the net effect of *F. prausnitzii* may be stage-dependent, with uncertain implications for late-stage ESCC.

Depletion of *F. prausnitzii* is a common feature across multiple GI malignancies and inflammatory bowel diseases, indicating it broadly reflects impaired intestinal health rather than an ESCC-specific alteration (Lin Y. et al., 2025; Shi et al., 2025b). This limits its utility as a diagnostic biomarker for malignant transformation. However, its reduction reflects compromised intestinal microecological homeostasis, positioning it as a candidate for microecological therapies (Singh et al., 2022; Formiga and Sokol, 2025). By reinforcing the intestinal barrier, *F. prausnitzii* confers protective benefits against ESCC progression by restricting the aforementioned endotoxin-driven systemic inflammation along the gut-esophageal axis (Singh et al., 2022; Shi et al., 2025b; Formiga and Sokol, 2025).

#### 3.3.2 Lachnospira

Members of the Lachnospiraceae family represent core commensals for intestinal homeostasis. As a representative genus within this family, *Lachnospira* is depleted in fecal samples from ESCC patients. This depletion occurs in parallel with broader gut dysbiosis and impaired metabolic-immune crosstalk (Deng et al., 2021; Cheung et al., 2022a; Shen et al., 2021). Preclinical evidence derived from genus-focused studies supports that *Lachnospira* exerts anti-tumor effects via four synergistic mechanisms. These include SCFA generation, TME remodeling, mucosal barrier reinforcement, and oncogenic signaling inhibition (Lavelle et al., 2022; Fan et al., 2023).

Similar to *F. prausnitzii, Lachnospira*-derived SCFAs suppress oncogenic signaling and induce apoptosis via universal HDAC inhibition, alongside AMPK pathway activation (Tier D; original model: non-EC *in vitro*). Butyrate produced by *Lachnospira* promotes dendritic cell (DC) maturation and CD8^+^ T cell infiltration while concurrently reducing immunosuppressive populations such as myeloid-derived suppressor cells (MDSCs) in preclinical models. Furthermore, its metabolites strengthen epithelial tight junctions and boost mucosal defenses to prevent pathogen translocation. These SCFA-mediated barrier reinforcements and immune modulations represent indirect, microbiota-wide protective effects, whereas the suppression of oncogenic signaling constitutes a direct anti-tumor mechanism (Lavelle et al., 2022; Fan et al., 2023).

The abundance of *Lachnospira*, alongside other Lachnospiraceae commensals, is elevated in individuals with balanced, fiber-rich nutrition (Nepal et al., 2025; Zünd et al., 2025). Dietary modulation targeting Lachnospiraceae enrichment has been proposed to alleviate GI toxicity induced by chemoradiotherapy (Cheatham et al., 2025). However, clinical evidence validating this hypothesis remains scarce (Nasrah et al., 2025), limiting translational application. These barrier-protective and immunomodulatory effects confer indirect benefits against ESCC progression via the aforementioned gut-esophageal axis (Cheatham et al., 2025). Diet-associated abundance shifts are not unique to *Lachnospira*, reflecting the multifactorial nature of gut microbial dynamics (Facciotti et al., 2025). Furthermore, SCFAs do not universally exert host-protective effects. While SCFAs maintain immune homeostasis under physiological conditions, elevated butyrate has been linked to impaired responses to cytotoxic T-lymphocyte-associated protein 4 (CTLA-4) blockade in preclinical models, indicating context-dependent functional outcomes (Dayhimi et al., 2025; Lim et al., 2024).

## 4 Clinical application value of gut microbiota in EC

Translational research on gut microbiota in EC mainly focuses on two directions: prognostic prediction and microbiota-targeted therapy. For predictive applications, microbial signatures serve as non-invasive biomarkers to stratify treatment response and clinical outcomes (Table 3). Current studies primarily focus on immune checkpoint inhibitor (ICI) and radical chemoradiotherapy efficacy prediction, with limited data on long-term survival.

**Table 3:** Gut microbial biomarkers for predicting treatment response and prognosis in esophageal cancer.

Clinical scenario	Microbial biomarker	Abundance-outcome association (with brief mechanistic hint)	Evidence strength
Post-op recurrence/survival	*Butyricimonas*	High abundance correlates with early recurrence and mortality (may expand Tregs and induce immunosuppression).	†
Post-op survival	*Bacillus* spp.	High abundance correlates with favorable OS and CSS (enhances intestinal barrier and innate immunity via exopolysaccharides).	†
Post-op survival	*Proteus*	Presence correlates with worse OS and RFS (activates LPS-TLR/NF-κB cascade and promotes toxic secondary bile acids).	†
nICRT response (pCR)	*SCFA* producers (*Faecalibacterium, Dialister, R. inulinivorans*)	High baseline abundance correlates with pCR (promotes DC maturation, antigen presentation, and CD8^+^ T infiltration).	†
nICRT resistance	*Escherichia-Shigella, Veillonella*	High abundance correlates with non-pCR (excessive TLR4/NF-κB activation and immunosuppressive cell recruitment).	†
Anti-PD-1 + chemo resistance	*Ligilactobacillus salivarius*	High abundance correlates with poor response (ILA metabolite activates AhR, driving CD8^+^ Tpex cell exhaustion).	‡
TP chemotherapy response	*B. vulgatus, B. ovatus*	High baseline abundance correlates with better response (SCFA-mediated epigenetic regulation and Warburg effect reversal).	†
TP chemo adverse events	*B. uniformis, B. ovatus*	Overabundance correlates with fewer high-grade AEs (reduced systemic spread of pro-inflammatory metabolites).	†

OS, overall survival; CSS, cancer-specific survival; RFS, recurrence-free survival; pCR, pathological complete response; nICRT, neoadjuvant immunochemoradiotherapy; TP, paclitaxel + cisplatin; AEs, adverse events. Quantitative thresholds: A preoperative Butyricimonas relative abundance >0.75% is identified as a high-risk marker for early recurrence. Other specific quantitative thresholds and cohort inclusion criteria are detailed in the primary literature to maintain table conciseness.

†, Hypothesized from indirect evidence; ‡, supported by direct experimental evidence. CD8^+^ T, cytotoxic T lymphocytes; MDSC, myeloid-derived suppressor cells; TME, tumor microenvironment.

SCFA-producing commensals consistently correlate with better chemoradiotherapy responses, while pathobionts (Proteus, Escherichia-Shigella) and immunosuppressive strains (L. salivarius) drive poor prognosis and treatment resistance; however, current evidence relies heavily on retrospective single-center cohorts, severely limiting clinical translation as standalone predictive biomarkers.

Regarding intervention strategies, modalities can be conceptually stratified into three tiers based on their level of precision and mechanistic definition (Lin A. et al., 2025). Coarse modulation, exemplified by FMT, is positioned at the upper tier of clinical efficacy; however, it remains hampered by a “black box” mechanism of action and unresolved safety concerns, including pathogen transmission. Intermediate modulation utilizes live biotherapeutics and engineered strains, such as *Ligilactobacillus salivarius*, which produces indole-3-lactic acid (ILA) to suppress anti-tumor immunity (Zhou et al., 2026). This tier emphasizes a necessary transition from genus-level correlations to strain-level specificity. Ultimately, fine modulation involves postbiotics and microbial metabolites (e.g., direct supplementation with butyrate or targeted activation of cognate receptors like GPR43 or AhR), which circumvent the pharmacokinetic and safety challenges associated with stable live bacterial engraftment (Figure 4, Table 4).

**Figure 4:**
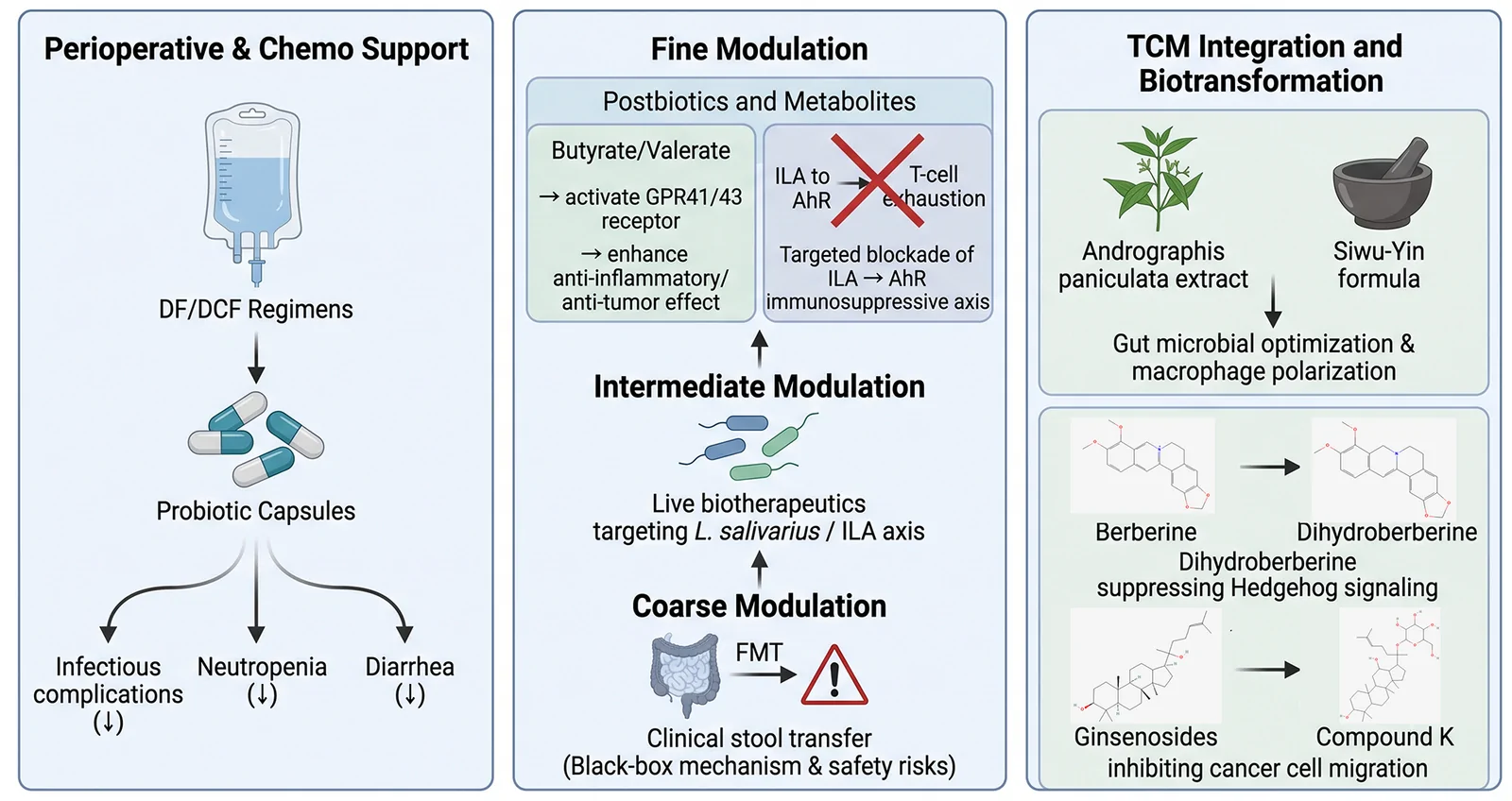
Strategies and molecular mechanisms of microbiota-targeted interventions in EC. **(Left)** Synbiotics/probiotics mitigate chemoradiotherapy-associated toxicity. **(Middle)** A three-tier precision framework for microbial modulation: Coarse (FMT), Intermediate (live biotherapeutics), and Fine (postbiotics/metabolite receptor activation). **(Right)** TCM integration entails both macroscopic ecological restoration and microscopic bidirectional biotransformation into active derivatives. PF, cisplatin plus 5-fluorouracil; DCF, docetaxel, cisplatin, and 5-fluorouracil; FMT, fecal microbiota transplantation; TCM, Traditional Chinese Medicine; ESCC, esophageal squamous cell carcinoma; ILA, indole-3-lactic acid; AhR, aryl hydrocarbon receptor.

**Table 4:** Microbiota-targeted interventions for esophageal cancer: strategies and clinical evidence.

Intervention type	Applicable scenario	Core clinical benefit	Evidence level
Synbiotics/Probiotics	Neoadjuvant chemotherapy (DF/DCF regimens)	Reduces grade 3 GI toxicity, febrile neutropenia, and severe diarrhea; maintains chemotherapy dose intensity.	Phase II RCT
Synbiotics/Probiotics	Post-esophagectomy	Shortens SIRS duration, lowers bacteremia and antibiotic-associated diarrhea, accelerates GI peristalsis recovery.	Phase II RCT
FMT (from ICI responders)	ICI-refractory advanced EC	Reverses anti-PD-1 resistance; promotes CD8^+^ T infiltration and pro-inflammatory cytokine release (DCR 46.2%).	Phase I/II
FMT (allogeneic)	Chemotherapy for advanced gastroesophageal cancer	Restores microbial diversity, reverses chemo-induced dysbiosis, and significantly improves disease control rate.	Phase II RCT
TCM: *Andrographis paniculata* extract	Palliative care (metastatic ESCC)	Enriches beneficial commensals, optimizes microbial diversity, and inhibits metastasis in a microbiota-dependent manner.	Phase II
TCM: Siwu-Yin formula	Chemoprevention (precancerous lesions)	Elevates SCFA and bile acid-metabolizing bacteria; induces macrophage polarization toward an anti-tumor phenotype.	Preclinical
TCM: Core monomers (Berberine, Ginsenosides)	Adjunctive therapy	Gut microbiota metabolize monomers into active derivatives (dihydroberberine, compound K) to enhance anti-tumor potency.	Preclinical

Evidence level indicates the highest level available. General safety and limitations: Probiotics carry bacteremia risk in immunocompromised patients; FMT carries risks of infection and adverse GI events, with undefined donor selection criteria; TCM studies largely lack chemical fingerprinting and standardized quality control. FMT, fecal microbiota transplantation; ICI, immune checkpoint inhibitor; RCT, randomized controlled trial; TCM, Traditional Chinese Medicine; SIRS, systemic inflammatory response syndrome; GI, gastrointestinal; DCR, disease control rate.

RCTs support probiotics for symptom management and FMT for overcoming ICI resistance, showing promising translational potential; however, the field lacks large-scale harmonized protocols, and most TCM evidence remains preclinical, representing a major bottleneck for clinical implementation.

While these microbiota-targeted therapies present theoretical promise, current literature frequently overstates their immediate clinical readiness. A balanced assessment of the translational pipeline must acknowledge that most studies remain early-phase, single-center, and small-sample.

First, regulatory frameworks for live biotherapeutic products (LBPs) remain fundamentally ambiguous. Unlike conventional drugs, LBPs are dynamic living consortia whose composition, viability, and functional output vary with manufacturing conditions. The FDA and EMA have not yet established standardized safety and efficacy approval criteria for complex microbial consortia, and the lack of validated potency assays and release specifications complicates clinical trial design and long-term safety monitoring (Barberio, 2024). This regulatory uncertainty substantially increases the time and cost of clinical development.

Second, severe logistical limitations hinder industrial scale-up. The strict anaerobic requirements of most core gut commensals demand highly complex manufacturing processes. Maintaining strain viability requires specialized cold-chain transportation and storage infrastructure, which compromises product consistency and limits widespread distribution. For FMT specifically, donor screening, pathogen testing, and batch-to-batch standardization remain unresolved, and the lack of unified donor selection criteria contributes to divergent efficacy across studies (Yadegar et al., 2024).

Third, prohibitive economic barriers prevent broad clinical integration. Precision microecological interventions, particularly tailored FMT, require personalized donor-recipient matching and continuous high-throughput multi-omics profiling. These procedures incur extreme costs currently unfeasible for routine clinical care. Moreover, health-economic evaluations of microbiota-targeted interventions in EC are almost entirely lacking, making it impossible to assess cost-effectiveness relative to standard therapies. Without reimbursement pathways, even effective interventions may fail to achieve clinical adoption (Yadegar et al., 2024).

Fourth, patient heterogeneity and biomarker validation remain major challenges. Baseline microbiome composition, host genetics, diet, medication use, and disease stage all influence treatment response. Current predictive models suffer from overfitting, lack external validation, and show suboptimal individual diagnostic performance compared with established serum assays (Zhao, 2026; Jiang et al., 2024; Nojima et al., 2026). The development of companion diagnostics for microbiota-based therapies is still in its infancy, and no validated biomarker has yet been approved for clinical use in EC.

Finally, the translational pipeline itself is long and attrition-prone. Preclinical findings in mouse models frequently fail to replicate in humans, and the causal contribution of specific taxa or metabolites to EC progression remains unproven in most cases. Even if a microbial intervention shows efficacy in a phase II trial, the probability of successful phase III confirmation and regulatory approval is low. Therefore, claims regarding the near-term clinical translation of microbiota-targeted therapies must be critically re-evaluated, and future research should prioritize rigorous, multicenter, randomized controlled trials (RCTs) with standardized protocols, validated biomarkers, and health-economic endpoints (Sugimoto et al., 2023; Shi et al., 2022).

### 4.1 Predictive value of gut microbiota as biomarkers for postoperative prognosis in EC

Gut dysbiosis is linked to postoperative outcomes in EC patients. Altered abundances of specific bacterial genera and species serve as non-invasive biomarkers for predicting postoperative recurrence and long-term survival.

Otsuka et al. (2024) reported that preoperative abundances of *Butyricimonas* and *Actinomyces* were higher in ESCC patients who developed postoperative recurrence. A preoperative *Butyricimonas* abundance exceeding 0.75% correlated with increased postoperative mortality and early recurrence. The genus exerts concentration-dependent dual effects via its major metabolite, butyrate (Yang et al., 2025). While butyrate possesses the aforementioned anti-tumor epigenetic functions, its capacity to expand regulatory T cells (Tregs) can confer immunosuppressive effects (Simpson et al., 2022; Coutzac et al., 2020). The drivers of postoperative recurrence involve minimal residual disease, surgical stress, and intestinal barrier injury, differing pathophysiologically from the immunotherapy context. The immunomodulatory properties of butyrate indicate a role in shaping postoperative immune reconstruction and influencing recurrence risk. However, the causal link between *Butyricimonas*-derived butyrate and poor postoperative prognosis requires validation in dedicated postoperative cohorts.

Maruyama et al. (2022) evaluated the prognostic value of gut microbiota in an ESCC cohort. Preoperative fecal *Bacillus* was an independent predictor of favorable overall survival (OS) and cancer-specific survival (CSS). The presence of *Proteus* correlated with worse OS and recurrence-free survival (RFS). Multivariate analysis confirmed *Proteus* as an independent risk factor for reduced RFS. Mechanistically, *Bacillus* strengthens the intestinal barrier and innate immunity to suppress pathogens, simultaneously secreting exopolysaccharides with anti-inflammatory and anti-tumor activities (Piewngam et al., 2018; Mohamed et al., 2021). In contrast, *Proteus* promotes the accumulation of toxic secondary bile acids and drives disease progression via the aforementioned LPS/TLR4/NF-κB inflammatory cascade (Tier D; original model: ESCC cell lines/EAC models) (Weh et al., 2024). These studies demonstrate that gut microbial signatures serve as non-invasive biomarkers for EC postoperative prognosis, providing a basis for risk stratification and microbiota-guided personalized therapy. Large-scale multicenter prospective cohorts with standardized sampling and bioinformatic pipelines are required to validate their clinical utility and cross-population applicability.

### 4.2 Predictive role of gut microbiota in the efficacy of neoadjuvant combination therapy for EC

#### 4.2.1 Neoadjuvant chemoradiotherapy combined with immunotherapy

Clinical comparisons of the gut microbiota between patients with and without pathological complete response (pCR) after nICRT have identified distinct microbial taxa as predictive biomarkers and potential therapeutic targets. Patients achieving pCR harbor elevated abundances of short-chain fatty acid (SCFA)-producing commensals, including *Faecalibacterium, Dialister*, and *Roseburia inulinivorans*. Through the aforementioned SCFA-mediated barrier reinforcement and TME remodeling, these microbes increase tumor infiltration of cytotoxic CD8^+^ T cells. The regulation of tumor cell programmed death-ligand 1 (PD-L1) expression by SCFAs is contested. Studies report both upregulation and downregulation in a concentration- and cell type-dependent manner. SCFA-mediated immune modulation enhances the anti-tumor efficacy of ICIs. The role of dietary fiber intervention in optimizing the nICRT response requires validation in prospective RCTs.

Conversely, patients without pCR exhibit gut dysbiosis characterized by the enrichment of pro-inflammatory pathobionts such as *Escherichia-Shigella*, alongside variable shifts in *Veillonella* and *Streptococcus*. The functional roles of *Veillonella* and *Streptococcus* are species- and context-dependent. Specific strains are associated with improved ICI response and enhanced CD8^+^ T cell infiltration in other tumor types. Their enrichment in nICRT non-responders likely reflects shifts in specific species within these genera rather than universal pathogenicity. *Escherichia-Shigella* and other pathobionts activate the aforementioned TLR4/NF-κB cascade, recruiting immunosuppressive cells to compromise nICRT efficacy (Tier D; original model: non-EC/*in vitro*) (Liu et al., 2024; van den Ende et al., 2024; Shaikh et al., 2024; Xu et al., 2022). Recent studies reveal layered regulatory mechanisms beyond the SCFA pathway: genotoxins and toxic secondary bile acids from pathogens aggravate immunosuppression, whereas immunomodulatory sphingolipids derived from commensals strengthen anti-tumor immunity (Yang et al., 2025). Variations in sampling protocols and patient backgrounds yield inconsistent results for certain taxa. However, multiple studies confirm that the enrichment of SCFA-producing bacteria correlates with better nICRT outcomes (Liu et al., 2024; van den Ende et al., 2024; Shaikh et al., 2024; Xu et al., 2022).

The preceding findings focus on radiotherapy-containing nICRT regimens. A separate line of research explores neoadjuvant anti-programmed cell death protein 1 (anti-PD-1) combined with chemotherapy (paclitaxel plus cisplatin) for ESCC. Zhou et al. (2026) delineated a microbiota-metabolite-immune axis governing treatment response in this radiotherapy-free regimen. By integrating four clinical cohorts, humanized microbiota mouse models, single-cell sequencing, and multi-omics data, the researchers found that *Ligilactobacillus salivarius* was enriched in patients with poor response to anti-PD-1 plus chemotherapy. Mechanistically, ILA produced by *L. salivarius* activates the aryl hydrocarbon receptor (AhR) and inhibits NF-κB signaling. This pattern differs from the TLR4/NF-κB activation observed in nICRT non-responders. It induces terminal differentiation of NKG7^+^CD8^+^ progenitor-exhausted T (Tpex) cells into terminally exhausted T (Tex) cells, thereby blunting systemic anti-tumor immunity. The abundance of *L. salivarius*, serum ILA levels, and intratumoral NKG7^+^CD8^+^ Tpex cell proportion were validated as independent predictive biomarkers. These variables demonstrated predictive efficacy surpassing that of PD-L1 expression, prompting proposals for corresponding targeted interventions.

In contrast, commensals including *Akkermansia* and specific *Bacteroides* species were enriched in immunotherapy responders. The *Bacteroides* genus exhibits functional heterogeneity across species. Certain members compromise barrier integrity and promote inflammation in postoperative settings. Conversely, specific strains enhance anti-tumor immunity in the context of ICIs. These findings highlight the context-dependent effects of the gut microbiota. Microbial regulation varies between radiotherapy-containing regimens and chemotherapy-alone regimens. Furthermore, microbial metabolites modulate the immunotherapy response via distinct signaling pathways (Figure 3).

#### 4.2.2 Neoadjuvant chemoradiotherapy

The gut microbiota modulates the efficacy of neoadjuvant chemoradiotherapy (nCRT) in EC via three pathways. These include TME remodeling, host metabolic reprogramming, and inflammatory regulation. Clinical and preclinical studies verify the predictive value of microbial signatures for nCRT response, with distinct taxa exerting opposing effects on chemoradiosensitivity.

Sasaki et al. (2023) identified differential gut microbial profiles between nCRT-sensitive and resistant ESCC patients. Lactobacillaceae and Streptococcaceae were enriched in responders. Lactobacillaceae modulates intestinal immune homeostasis and enhances mucosal barrier integrity primarily via lactic acid production, which supports CD8^+^ T cell infiltration and alleviates systemic inflammation. Certain commensal Streptococcaceae strains are associated with enhanced treatment response, potentially via the activation of interferon-gamma (IFN-γ) signaling to support antigen-presenting cell (APC) function. In contrast, *Fusobacterium nucleatum*, a representative pathogenic species within the *Fusobacteriaceae* family enriched in locally advanced and metastatic EC, has been associated with treatment resistance. In CRC models, its surface adhesin A (FadA) binds host E-cadherin and activates β-catenin signaling, promoting tumor proliferation and invasion (Tier D; original model: CRC); whether this mechanism operates in EC is unknown. In addition, *F. nucleatum* may induce chronic systemic inflammation and disrupt immune homeostasis, contributing to an immunosuppressive TME (Sasaki et al., 2023).

Li et al. (2021) confirmed SCFA-producing bacteria as predictive biomarkers for EC nCRT. Baseline *Roseburia* abundance positively correlated with chemotherapy response, presenting higher levels in responders. Beyond baseline SCFA-mediated epigenetic regulation, *Roseburia* specifically downregulates the VEGF-HIF-1α angiogenic pathway and promotes CD8^+^ T/NK cell activation to enhance chemosensitivity. Receiver operating characteristic (ROC) analysis identified a 21.2% reduction in *Roseburia* abundance post-chemotherapy as the cut-off value for predicting disease progression, with detailed diagnostic performance metrics reported in the original study (Li et al., 2021).

However, *Blautia* yields conflicting results. One study reported that elevated *Blautia* abundance at baseline before neoadjuvant chemotherapy was associated with reduced chemosensitivity in EC (Hasuda et al., 2023). Conversely, studies on other GI cancers reported favorable progression-free survival in patients with high *Blautia* levels (Martini et al., 2022; Wang B. et al., 2024). These discrepancies indicate that *Blautia* function is cancer-type specific, requiring mechanistic research in the setting of EC nCRT.

In a cohort of advanced ESCC patients receiving paclitaxel plus cisplatin chemotherapy, Li et al. (2024) demonstrated that gut microbial signatures predict treatment efficacy and chemotherapy-related toxicity. Higher baseline levels of *Bacteroides vulgatus* and *Bacteroides ovatus* were associated with better treatment response. Conversely, *Bacteroides uniformis* and *Bacteroides ovatus* correlated with high-grade adverse events. The dual association of *B. ovatus* with both efficacy and toxicity is potentially explained by strain-level heterogeneity, abundance-dependent effects, or interactions with concurrent microbial taxa. A combined model based on these taxa achieved high predictive performance for toxicity [area under the curve (AUC) > 0.8]. Mechanistically, commensals such as *Bacteroides* spp. improve chemosensitivity by modulating bile acid metabolism, reversing the Warburg effect, and restricting inflammation-driven resistance via competitive exclusion and barrier reinforcement. Conversely, overabundant pathobionts exacerbate toxicity by accelerating the systemic spread of drug metabolites and endotoxins through impaired mucosal barriers.

Gut microbial signatures serve as non-invasive biomarkers for nCRT efficacy and toxicity in EC, providing a basis for microbiota-guided neoadjuvant therapy. Most available evidence derives from retrospective single-center cohorts with small sample sizes, lacking unified standards for sample collection, sequencing, and bioinformatic analysis. Multicenter prospective studies are required to validate these findings and develop clinically applicable microbial risk stratification models.

### 4.3 Clinical application and mechanism of gut microbiota-targeted intervention in EC therapy

#### 4.3.1 Applications of microecological preparations in optimizing treatment outcomes and reducing postoperative complications

Chemotherapy, radiotherapy, and surgical trauma induce gut dysbiosis in EC patients. This dysbiosis contributes to postoperative infection, GI toxicity, and poor treatment tolerance. Clinical evidence confirms that microecological preparations, specifically synbiotics, restore gut homeostasis, elevate SCFA concentrations, maintain intestinal barrier integrity, and suppress pathogenic overgrowth, yielding protective effects (Fukaya et al., 2021; Sugimoto et al., 2023; Motoori et al., 2017, 2022; Tanaka et al., 2012; Yokoyama et al., 2014).

Clinical trials verify that peri-chemotherapy synbiotic supplementation reduces infectious complications and GI toxicity in EC patients receiving neoadjuvant chemotherapy. The protective effects vary by chemotherapy regimen and drug intensity. For patients treated with cisplatin plus 5-fluorouracil (PF), synbiotics reduce bacterial translocation, bacteremia, and grade 3 GI toxicity (Fukaya et al., 2021). For patients receiving the docetaxel, cisplatin, and 5-fluorouracil (5-FU) regimen, synbiotics combined with enteral nutrition correlated with reduced febrile neutropenia (*P* = 0.087). This intervention also decreases grade 4 neutropenia and grade 2–4 diarrhea. The combination therapy helps patients maintain a higher relative dose intensity of chemotherapy and improves treatment tolerance. Improved treatment tolerance correlates with better long-term survival (Sugimoto et al., 2023; Motoori et al., 2017, 2022).

Synbiotics demonstrate protective effects after esophagectomy. Esophagectomy combined with extensive lymphadenectomy disrupts the gut microbiota and intestinal barrier, elevating the risk of postoperative complications. Postoperative synbiotic use shortens the duration of systemic inflammatory response syndrome (SIRS), lowers the incidence of bacteremia and antibiotic-associated diarrhea, and accelerates the recovery of GI peristalsis (Sugimoto et al., 2023; Motoori et al., 2017). Mechanistically, probiotics and prebiotics work synergistically to enrich taxa such as *Bifidobacterium* and *Lactobacillus*. Through cross-feeding interactions, these microbes boost the abundance of SCFA-producing commensals, which subsequently alleviate treatment-related complications via the aforementioned barrier-fortifying and anti-inflammatory pathways (Fukaya et al., 2021; Sugimoto et al., 2023; Motoori et al., 2017, 2022; Tanaka et al., 2012; Yokoyama et al., 2014).

Microecological preparations provide supportive care for EC patients during neoadjuvant chemotherapy and the perioperative period. Targeting the gut-immune axis, they reduce complications and optimize treatment outcomes. Most existing studies are single-center trials with heterogeneous interventions, including variable strains, dosages, and treatment courses. Multicenter RCTs with standardized protocols are required to define optimal application strategies.

#### 4.3.2 Mechanisms and clinical research of FMT in regulating chemotherapy and immunotherapy efficacy for EC

FMT is a microbiota-targeted approach that remodels gut microecology to enhance anti-tumor immunity. It reverses ICI resistance and improves therapeutic outcomes in EC. Preclinical and early clinical data indicate its capacity to increase chemosensitivity.

In the context of immunotherapy, clinical progress has been achieved in FMT plus ICI therapy for advanced EC. A phase I-II trial evaluated the regimen of antibiotic preconditioning, FMT, and ICI (A-FMT) in patients with refractory advanced EC. This study confirmed that microbial reconstitution reinvigorates systemic anti-tumor immunity by promoting cytotoxic CD8^+^ T cell infiltration following PD-1/PD-L1 blockade (Yoshinami et al., 2025). While direct validation in this cohort is pending, proposed mechanisms include cross-reactive T cell activation via molecular mimicry and the aforementioned SCFA-mediated immune modulation (Yoshinami et al., 2025). Furthermore, a prospective single-arm study evaluating FMT (derived from ICI responders) in ICI-resistant advanced solid tumors, including ESCC, demonstrated a reversal of anti-PD-1 resistance with a disease control rate (DCR) of 46.2% (Kim et al., 2024). Enrichment of specific taxa, such as *Bifidobacterium* and *Akkermansia muciniphila*, is hypothesized to drive this crucial TME remodeling (Kim et al., 2024). However, these early-phase studies face limitations, notably small sample sizes, single-center designs, and the lack of unified donor selection criteria, which frequently leads to divergent efficacy.

FMT also modulates the chemotherapy response in EC. A phase II randomized, double-blind controlled trial using autologous FMT as the control evaluated patients with advanced GI malignancies, with the majority diagnosed with EC (de Clercq et al., 2021). Patients receiving allogeneic FMT achieved a higher DCR than the control group, correlating with prolonged median overall survival (mOS). This provides evidence supporting a chemosensitizing effect of microbial intervention in upper GI cancers, though these findings require validation in larger confirmatory trials (de Clercq et al., 2021). The underlying mechanisms remain under investigation, with hypotheses pointing toward the reversal of chemotherapy-induced dysbiosis, the aforementioned SCFA-mediated barrier preservation (Tier C/D; original model: mixed preclinical/clinical evidence), and the modulation of tumor metabolism to enhance drug cytotoxicity (de Clercq et al., 2021). No single microbial species was identified, indicating that FMT efficacy relies on the overall functional output of the microbial community. Future trials must prioritize FMT combined with neoadjuvant therapy for resectable EC to improve chemoradiosensitivity and curative resection rates. Multi-omics and ecological network analyses will be critical to decipher these interactions and standardize FMT application.

In addition to donor heterogeneity, undefined intervention timing is an obstacle for standardized FMT use. Multiple intervention windows have been proposed; however, no consensus has been reached (Yadegar et al., 2024; Muller et al., 2026; Halsey et al., 2023; Baruch et al., 2021). The pre-neoadjuvant window (1–2 weeks before treatment) is proposed as a potential timing based on mechanistic rationale. Early microbial remodeling establishes a tumor-suppressive immune microenvironment and improves subsequent treatment response (Yadegar et al., 2024; Halsey et al., 2023). Conversely, concurrent FMT is vulnerable to chemotherapy-induced microbial disruption, leading to less stable outcomes (Yadegar et al., 2024; Muller et al., 2026). FMT for ICI-resistant patients during the salvage window produces limited benefits. Perioperative and palliative FMT primarily reduce complications and improve quality of life, demonstrating minimal anti-tumor effects (Yadegar et al., 2024; Halsey et al., 2023; Baruch et al., 2021). Direct head-to-head comparisons of FMT timing strategies in EC are currently lacking. Consequently, consensus on optimal timing remains to be established. Standardized timing strategies and individualized regimens are required to address inconsistent efficacy across studies.

#### 4.3.3 Gut microbiota: the core mediator of TCM for anti-ESCC effects and microecological remodeling

TCM is applied in the management of GI cancers. Evidence indicates that the gut microbiota acts as a core mediator of TCM anti-ESCC effects, representing a mechanism distinct from conventional protein- and gene-based research paradigms (Yue et al., 2019; Feng et al., 2023; Wei et al., 2024). Both TCM monomers and compound formulas remodel microbial composition, reverse EC-associated dysbiosis, and restore gut homeostasis (Cheung et al., 2020; Chiu et al., 2023; Shi et al., 2022). For example, the water extract of *Andrographis paniculata* enriches beneficial commensals, depletes tumor-related taxa, optimizes microbial diversity, and inhibits tumor metastasis in a gut microbiota-dependent manner (Chiu et al., 2023; Cheung et al., 2022b). The formula Siwu-Yin elevates SCFA-producing and bile acid-metabolizing bacteria, modulates bile acid metabolism, and induces macrophage polarization toward an anti-tumor phenotype, suppressing esophageal precancerous lesions (Halsey et al., 2023). TCM has been proposed to inhibit pathobionts such as *Fusobacterium nucleatum* to suppress the aforementioned TLR4/NF-κB cascade, although this is largely inferred from non-EC models or *in vitro* studies (Tier D/E; original model: *in vitro*/non-EC). Concurrently, TCM has also been reported to upregulate SCFA-producing genera (*Faecalibacterium, Bifidobacterium*) and beneficial microbes including *Lactobacillus* and *Akkermansia* (Yue et al., 2019; Feng et al., 2023; Qiao et al., 2025). By enriching these specific taxa, TCM confers indirect anti-tumor benefits via the previously established barrier-fortifying and endotoxin-restricting pathways without necessitating direct cytotoxic action (Yue et al., 2019; Wei et al., 2024; Qiao et al., 2025). Conversely, the gut microbiota metabolizes TCM components into active derivatives.

Berberine is converted to dihydroberberine, suppressing Hedgehog signaling, whereas ginsenosides are transformed into compound K, inhibiting ESCC migration. These transformations improve drug bioavailability and anti-tumor potency (Yue et al., 2019; Wei et al., 2024). Bidirectional interactions between TCM and the microbiota underpin the multi-target synergistic effects of TCM, differing from single-agent targeted therapy (Yue et al., 2019; Qiao et al., 2025). As a low-toxicity supplementary therapy, TCM is suited for advanced ESCC patients, mitigating the gut homeostasis disruption caused by conventional chemotherapy (Yue et al., 2019; Wei et al., 2024). Restoring gut eubiosis is a shared anti-tumor mechanism across GI cancers, revealing a dimension of TCM efficacy overlooked in conventional research (Cheung et al., 2020; Chiu et al., 2023; Feng et al., 2023; Shi et al., 2022). In this context, the gut microbiota bridges TCM philosophy and modern microbial ecology, providing a direction for personalized ESCC therapy and integrative oncology (Yue et al., 2019; Wei et al., 2024; Qiao et al., 2025).

Nevertheless, this field presents methodological limitations requiring cautious interpretation. Most studies observe phenotypic changes induced by complex compound formulas, lacking rigorous molecular pharmacological validation. Mechanistic inferences often rely on crude extracts rather than purified bioactive monomers, such as flavonoids and polysaccharides. The absence of chemical fingerprinting and unified quality control causes batch-to-batch variation, impairing experimental reproducibility and hindering clinical translation. To establish causal evidence and meet regulatory requirements, future research must focus on identified active compounds, combine network pharmacology, and validate core signaling nodes, including STAT3 and AKT1, using germ-free animal models and clinical cohorts. Without mechanistic validation, TCM-microbiota interactions remain descriptive correlations rather than actionable therapeutic targets (Li, 2021; Heinrich et al., 2022).

## 5 Conclusions and future perspectives

In summary, while the gut-esophageal axis represents a highly promising frontier in EC research, the field must urgently transition from descriptive dysbiosis profiling to rigorous causal validation (O'Leary, 2021). Although mechanisms such as systemic inflammation and barrier disruption offer biological plausibility, their heavy reliance on non-EC models underscores a significant knowledge gap. Moving forward, acknowledging the dynamic influence of dietary context, host genetics, and treatment-induced microecological shifts will be essential for translating these microbial signals from correlative observations into robust clinical tools.

### 5.1 Early diagnostic potential

Fecal microbiota profiling represents a non-invasive strategy for early cancer detection, providing minimal invasiveness, convenient sampling, and patient compliance (Wong and Yu, 2023). Studies identify microbial signatures associated with EC and precursor lesions (Deng et al., 2021; Cheung et al., 2022a; Shen et al., 2021; Gao et al., 2024; Huang et al., 2024; Li Y. et al., 2025; Li et al., 2022). These biomarkers require systematic multicenter validation due to inter-study heterogeneity and inter-individual variability. Multi-omics integration combining microbial signatures with metabolomics, proteomics, and clinical data offers an approach toward robust, non-invasive EC early screening (Wong and Yu, 2023; Qu et al., 2023; Coker et al., 2022).

### 5.2 Therapeutic predictive and interventional value

Gut microbiota signatures predict EC treatment responses, though limitations persist. Candidate biomarkers exhibit overfitting, a lack of independent external validation, and suboptimal individual diagnostic performance compared with established serum assays. Inconsistent normalization pipelines, missing data handling protocols, low-abundance filtering criteria, and technical variability control undermine cross-study reproducibility. Adopting analytical frameworks derived from metabolomics research improves cross-study comparability and molecular reproducibility.

For clinical translation, while early-phase trials highlight the potential of microbiome modulation, significant hurdles remain. Future efforts must prioritize establishing unified donor selection criteria and optimal intervention timing for FMT (Lin A. et al., 2025; Yadegar et al., 2024). Furthermore, integrating multi-omics to dissect donor microbiota stability and exploring autologous FMT strategies are essential steps toward standardizing these therapies (Lin A. et al., 2025). Concurrently, validating the molecular targets of TCM compounds in microbiota-dependent models warrants further investigation to solidify its complementary role in EC management (Gou et al., 2023).

### 5.3 Key limitations of current research

Despite recent advances, several critical limitations hinder the clinical translation of microbiota-targeted strategies in EC.

First, current literature exhibits extreme geographic and pathological bias, focusing predominantly on East Asian ESCC cohorts with limited EAC data. This imbalance directly restricts the generalizability of microbial biomarkers and predictive models. The etiology, genomic landscape, and environmental risk factors of ESCC and EAC are fundamentally distinct (Thrift, 2021; Deboever et al., 2024). ESCC in East Asia is heavily influenced by specific dietary habits and local environmental exposures, whereas EAC in Western populations is primarily driven by obesity, gastroesophageal reflux disease, and Barrett's esophagus (Thrift, 2021). These divergent risk factors shape the gut microbiome differentially. Consequently, the microbial biomarkers, predictive models, and pathogenic mechanisms identified in Chinese ESCC cohorts (e.g., the *L. salivarius*/ILA/AhR axis) cannot be directly extrapolated to Western EAC populations. Treating these two distinct malignancies as a homogeneous entity in microbiota research constitutes a methodological flaw (Baba et al., 2025). Future studies must adopt stratified designs, report subtype-specific results, and validate candidate biomarkers in independent cross-regional cohorts.

Second, current evidence remains largely correlational, lacking causal proof linking dysbiosis to EC onset or progression. Nearly all ESCC microbiome studies are cross-sectional. Furthermore, mechanistic investigations heavily rely on the unverified extrapolation of generalized pro-inflammatory cascades (e.g., TLR4/NF-κB) from CRC models. Applying the evidence grading framework (Section 2.3), most microbiota-EC mechanisms currently fall within Tier C-D, with only isolated Tier A/B evidence. Disentangling causality requires three complementary strategies: prospective cohorts in EC-endemic regions with long-term follow-up; FMT experiments in germ-free or antibiotic-treated mouse models to establish direct causality; and single-strain colonization assays coupled with metabolite-host pathway analyses. Additionally, Mendelian randomization should be applied to mitigate reverse causation bias. Targets lacking sequential *in vivo* validation must be objectively classified as hypothesized mechanisms rather than established oncogenic drivers.

Third, the widespread absence of standardized methodological protocols generates substantial batch effects, restricting inter-study comparability. Achieving molecular reproducibility requires the urgent implementation of harmonized guidelines encompassing rigorous medication washout periods, standardized sampling, uniform 16S rRNA hypervariable region selection, and algorithm-based batch effect removal.

### 5.4 Multi-omics integration, spatial transcriptomics, and AI in EC microbiota research

Although 16S rRNA sequencing has established the taxonomic landscape of gut dysbiosis in EC, it cannot resolve functional gene expression, metabolite production, or spatial host-microbe interactions. Multi-omics approaches have therefore become essential to move from correlation to mechanism. Metagenomics provides strain-level functional potential, including virulence factors (e.g., *bft, pks* island) and metabolic gene repertoires (Makkar and Bishnupuri, 2025; Duan et al., 2024). Metatranscriptomics reveals which microbial genes are actively transcribed *in vivo*, distinguishing dormant colonists from functionally active pathobionts (Makkar and Bishnupuri, 2025). Metabolomics identifies microbially derived metabolites, such as SCFAs, secondary bile acids, indole derivatives, and polyamines, that directly modulate esophageal epithelial and immune cells (Duan et al., 2024). Proteomics and immunopeptidomics can map host-microbe protein interactions and antigen presentation, while single-cell transcriptomics resolves immune cell states (e.g., CD8^+^ Tpex vs. Tex) shaped by microbial signals (Le et al., 2025). Integrating these layers has already begun to clarify EC-relevant mechanisms. For example, Zhou et al. (2026) combined four clinical cohorts, humanized microbiota mouse models, single-cell sequencing, and multi-omics to delineate the *L. salivarius*/ILA/AhR/Tpex axis in anti-PD-1 resistance, providing EC-specific functional evidence (Tier A/B). Similarly, Liu et al. (2024) integrated microbiome and metabolome data to identify SCFA-producing commensals associated with nICRT response. These studies illustrate that multi-omics integration can elevate mechanistic claims from Tier C/D to Tier A/B, but such integration remains rare in EC research.

Two emerging technologies are particularly promising for closing remaining mechanistic gaps. First, spatial transcriptomics, when combined with single-cell sequencing and microbial fluorescence *in situ* hybridization (FISH), enables the precise physical localization of specific bacterial strains and their metabolites within the tumor microenvironment (Le et al., 2025). This is critical for distinguishing whether dysbiotic bacteria are merely luminal passengers or active mucosal invaders interacting directly with immune cell niches and malignant epithelia. In EC, spatial mapping could clarify whether translocated LPS or BFT acts locally at the esophageal tumor bed or systemically via the gut-esophageal axis, and whether microbial signals co-localize with specific immune subsets (e.g., Tregs, MDSCs, exhausted CD8^+^ T cells). Second, artificial intelligence (AI) and machine learning can decode high-dimensional multi-omics datasets, matching metagenomic functional repertoires with host spatial transcriptomics and systemic metabolomics (He et al., 2023). AI-driven network inference can identify core interactive nodes between microbiota and host immunity, prioritize causal candidates for functional validation, and build predictive models of treatment response. However, AI models require large, harmonized, multi-center datasets and rigorous external validation; otherwise, they risk overfitting and poor generalizability, a limitation already observed in current microbiota biomarker studies (Le et al., 2025).

Integrating multi-omics, spatial transcriptomics, and AI with the evidence grading framework proposed in Section 2.3 provides a rational path to causal inference. Specifically, multi-omics can generate mechanistic hypotheses; spatial transcriptomics can localize and validate these candidates *in situ*; AI can prioritize targets and predict responses; and EC-specific *in vivo* models (e.g., germ-free or antibiotic-treated EC mice, 4NQO-induced orthotopic models) can definitively provide Tier A causal evidence. Without this integrated pipeline, microbiota-EC associations will remain largely descriptive, and clinical translation will continue to lack a rigorous mechanistic rationale.

### 5.5 Future perspectives

Clinically, a synergistic strategy combining conventional anti-tumor therapy with microbiota-targeted interventions (including standardized FMT and mechanism-validated TCM) offers a promising paradigm for EC management (Fan, 2024; Zeng et al., 2024; Wang et al., 2020). However, recognizing the genotype-dependent metabolic heterogeneity of tumor cells, future research must move beyond one-size-fits-all sensitization approaches. The field must map the oncogenotype-metabolism-microbiota adaptive network, enabling precision microecological interventions tailored to specific tumor metabolic vulnerabilities, such as altered bile acid and SCFA metabolism (Chen et al., 2019). Ultimately, overcoming current technical and clinical barriers requires the integration of multi-omics, spatial transcriptomics, and AI technologies alongside rigorous *in vivo* causal validation using functional models (Makkar and Bishnupuri, 2025; Duan et al., 2024). A balanced, objective assessment of translational readiness, prioritizing standardized multicenter prospective cohorts (Lin et al., 2026) and unified operating procedures (Lyte et al., 2025), will be essential to transition gut microbiota research from correlative observations into actionable precision oncology.
